# Milk production profile of *Barela* camel (*Camelus dromedarius*) supplemented with postbiotics in a semi-intensive management system: pilot study

**DOI:** 10.3389/fvets.2025.1576912

**Published:** 2025-06-02

**Authors:** Asim Faraz, Hassan Qadir Buzdar, Abdul Waheed, Syeda Maryam Hussain, Sajjad Ur Rahman, Muhammad Mukarram Bashir, Barbara Padalino

**Affiliations:** ^1^Department of Livestock and Poultry Production, Bahauddin Zakariya University Multan, Multan, Pakistan; ^2^Department of Livestock Production and Management, Pir Mehr Ali Shah-Arid Agriculture University Rawalpindi, Rawalpindi, Pakistan; ^3^College of Veterinary Science, Multan University of Science and Technology, Multan, Pakistan; ^4^Bio Augment Research Laboratory Jaranwala Road, Faisalabad, Pakistan; ^5^Department of Agricultural and Food Sciences, University of Bologna, Bologna, Italy; ^6^Faculty of Science and Engineering, Southern Cross University, Lismore, NSW, Australia

**Keywords:** camel, dromedary, postbiotics, milk, semi-intensive

## Abstract

This pilot study investigated the effects of postbiotics supplementation on both milk yield and composition within a semi-intensive management system in *Barela* camels. The key indicators included daily milk yield, fats, protein, solid not fats, and lactose levels. A total of 12 dairy camels from early to mid-lactation stages (second to fourth parity) were divided into four groups to obtain similar milk production among groups. Prior to the study, all camels were dewormed and confirmed for good health status. The first group served as the control and was permitted to graze for only 8 h per day without any supplementation. The second group received an additional 3 kg of concentrate feed alongside the same grazing schedule. The third and fourth groups were supplemented with 3 kg of concentrate feed plus 6 grams and 15 grams of extra pure metabolites (postbiotics - XPM), respectively, while maintaining the daily grazing duration of 8 h. The trial spanned 45 days, with an initial adaptation period of 15 days. Milk yield was recorded at four intervals: days 0, 16, 30, and 45. Milk composition analysis occurred on days 0 and 45 to establish baseline and final metrics. A complete randomized design was used, and one-way ANOVA was applied for statistical analysis at a significance level of 5%. The least significant difference test facilitated comparisons among treatment means. Results indicated significant differences in milk production across groups (*p* < 0.005), with the highest yield observed in the fourth group (8.93 ± 0.74 kg) compared to the control group (4.64 ± 0.32 kg) (*p* = 0.0010). In terms of milk composition, there was a notable effect on fat percentage among treatment groups, with the fourth group exhibiting the highest fat content (3.40 ± 0.05%) and the control group showing the lowest (2.82 ± 0.05%) (*p* = 0.0450). However, variations in protein, lactose, and solids-not-fat levels were not statistically significant. In short, postbiotics significantly enhance fat content in *Barela* dromedary camels, highlighting their potential as a valuable dairy breed within semi-intensive management systems. This will serve as a pilot study for the field of camel science, which could be used for further detailed studies about camel semi-intensive and intensive feeding management systems.

## 1 Introduction

More than 50 distinct breeds of camels, totaling 38.5 million (Food and Agriculture Organization Corporate Statistical Database) (1, 2) are found globally and approximately 27 breeds are present in Africa, Pakistan, and Saudi Arabia (2–4). The dromedary camels (*Camelus dromedarius*) hold significant importance in sustaining life (5), as they are adapted to thrive in harsh environments, and limited water availability (6), such as *Cholistan* and *Thal*. This adaptability allows them to utilize poor-quality forage, making them indispensable for pastoral communities. The climatic factors and demographical situations influence the environment, feed and water availability, and feed quality, hence directly affecting camels’ milk (7) and meat production. Pakistan has more than 1.2 million camels (8), and the *Barela* breed is famous for its dairy potential (70). Generally, the milk yield in dromedaries varies between 3.5 and 20 liters, depending on geographical regions (9).

Camel milk is a primary product derived from camels, serving as a crucial dietary component in arid and semi-arid regions (10). The yield and composition of milk are influenced by various factors, such as breed, nutritional intake, physiological status, milking practices, milking frequency, calf suckling behavior, and feed and water availability (6, 11, 12). These variables collectively determine the quantity and quality of milk produced, making camel milk production a complex interplay of environmental, genetic, and management factors. The presence of immunoglobulins and other bioactive compounds in camel milk has great potential for health benefits, as anti-inflammatory (13, 14, 59, 61). However, the milk profile is closely linked to age, sex, and reproductive status. The milk composition of *Barela* typically contains higher fats and protein compared to cows, with average fat 4.26%, protein 3.62%, solid non-fat (9.02%), and total solids (13.28%) (15, 16). The camels’ milk has lower lactose levels (4.84%), making it suitable for individuals with lactose intolerance (7). *Barela* camel milk exceeds the nutritional standards set by other dromedary breeds globally (11). *Barela* can produce an average of 6.0-liter milk/day, with a 586-day lactation period and a lactation curve peaking up to the fourth month (6). This pattern is consistent across different breeds and management systems, indicating that dromedaries can sustain high levels of milk production under optimal conditions. Seasonal variations also play a crucial role in influencing milk production throughout the year (17). Thus we need to adopt management strategies to consider seasonal dynamics and plan feeding regimens.

Despite the recognized potential for camel milk production, several challenges impede optimal productivity. Limited research on camel management in Pakistan has resulted in a lack of standardized protocols for feeding and milking (16). Furthermore, factors as drought can significantly impact forage availability, affecting the camels’ health and productivity. Mustafa et al. (6) emphasized improved management practices for improvement in reproductive efficiency and productivity in dromedary camels, which can be achieved by regular health checks, vaccinations, breeding programs, and ensuring welfare.

Postbiotics were proposed in 2019 as “preparation of inanimate microorganisms and/or their components for improving animal’s health” by the International Scientific Association of Probiotics and Prebiotics (18). Their supplementation (19) has emerged as a promising strategy to enhance both milk yield and composition in animals. They promote a healthy gut environment, improve the nutrient digestibility and absorption (20), modulate the gut microbiota and inhibit pathogens, possibly via quorum sensing (cell–cell communication) and adhesion (21). Hence, leading to increased dry matter intake and efficient functioning of the gastrointestinal tract. Camel α-lactalbumin utilizes bioactivities for reactive oxygen species scavenging and anti-inflammation for treating specific metabolic disorders (22). These benefits translate to higher milk production, with a significant increase in milk yield and milk composition (fat, protein, and lactose) (23). Postbiotics enhance the colostrum quality for fresh lactating dairy camels and reduce the presence of undesirable compounds such as urea and ammonia-N production. They also reduce the greenhouse gas production, thus utilizing energy to improve the animal’s productivity.

Postbiotics have been proven to improve animal performance, but there are limited data on specific postbiotics for enhancing camels’ milk yield. Certain probiotics in camel milk produce postbiotics (24). *Lactobacillus brevis* strains synthesize *gamma-aminobutyric acid* (GABA), which can regulate blood glucose and reduce blood lipid levels (25), influencing the gut–brain axis and systemic metabolic health (26). Furthermore, some probiotic bacteria in camel milk produce exo-polysaccharides (EPSs). Certain lactic acid bacteria (LAB) such as *Lactobacillus plantarum*, *Leuconostoc mesenteroides*, and *Weissella* species, are present in raw camel milk, and *Lactobacillus paracasei*, in fermented camel milk products (27), have antagonistic properties (28). Though these findings are promising, more studies are needed to determine whether postbiotics can directly influence milk yield in camels.

Camels are often raised in marginalized communities, and their welfare standards as good feeding, are often compromised, and animals with low body condition scores are fed only on pasture (62). Consequently, the hypothesis supports that supplementation with postbiotics would increase milk quantity and quality, with improvement in camel welfare. This study aimed to compare the effects of an integration of specially formulated concentrate feed (Dairylac camel feed) and postbiotics (XPM) on the milk quantity and quality in camels kept under a semi-intensive management system.

## 2 Materials and methods

### 2.1 Animal ethics

This study was approved by the Department of Livestock and Poultry Production, Bahauddin Zakariya University Animal Ethics Committee (Protocol number 279/15-10-2024).

### 2.2 Methodology

This study was conducted at the camel facility in Jalalpur Pirwala, located in Chak 81 M, south Punjab, adjacent to the *Cholistan*, recognized as a significant hub for camel milk production and export to Gulf countries and China. The trial involved 12 female dairy camels, selected based on their parity (second to fourth) and lactation stage (early to mid) during November to December 2024. The camels were divided into four groups, with three replicates in each, having the same average milk (20 liters/group) production. The camels were kept at an average ambient temperature (AAT) between 24.6°C and 8°C during the day and night, average relative humidity (ARH) of 26%, and average day length (ADL) of 10 h and 18 min during the year’s shortest days, with sunrise at 6:52 a.m. and sunset at 5:26 p.m. (PKT). Thereby ensuring exposure to natural climatic variations that may affect milk production and quality.

**Figure 1:**
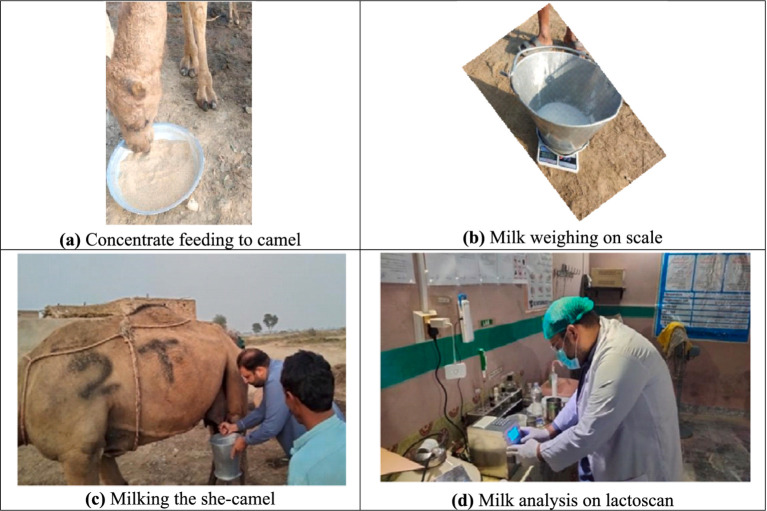
Different research activities during the experiment **(a)** concentrate feeding to the camel, **(b)** milk weighing on the scale, **(c)** milking of the she-camel, and **(d)** milk analysis on lactoscan.

All camels received treatment for endo and ecto-parasites before the trial to ensure their good health status. An adaptation period of 15 days preceded a 45-day trial where milk yield and composition were measured. The camels were provided with clean water bi-daily, commercial ration (Figure 1; Table 1) (29) and grazed for 8–10 h daily under consistent conditions. The feeding regimen varied by group: Group 1: Grazing only; Group 2: Grazing + 3 kg of concentrate; Group 3: Grazing + 3 kg of concentrate + 6 grams of XPM; Group 4: Grazing + 3 kg of concentrate + 15 grams of XPM (Procured from Bioaugment research laboratory, Faisalabad - Table 2).

**Table 1:** Chemical composition of the commercial ration offered to experimental camels.

No.	Parameters	Percentage
1	Dry matter	88%
2	Crude protein	16%
3	Crude fat	5.5%
4	Crude fiber	6.5%
5	Ash	9%
6	ME	12 MJ/Kg

**Table 2:** Chemical composition of XPM offered to experimental camels.

S. No.	Parameters	Percentage
1	Crude protein	15–16%
2	Crude fat	1–2%
3	Crude fiber	22–25%
4	Ash	7–9%
5	Saccharomyces yeast and the media consisting of soyhulls, wheat bran, and cane molasses	

### 2.3 Parameters

Milk yield was recorded at days 0, 16, 30, and 45; two times in the morning (6 a.m. and 8 a.m.) and evening (6 p.m.) (Figure 1). Milk composition was analyzed only at days 0 and 45 with Lactoscan equipment (Milkotronic Ltd., China). Daily AAT, ARH, ADL, and grazing patterns were documented, considering environmental factors affecting milk production. The concentrate feed for each group was measured and provided daily to guarantee precision in supplementing, and the XPM dosages were adjusted to align with experimental specifications, with rigorous compliance with feeding schedules. This analysis aimed to provide insights into cost-effective feeding strategies for camel milk producers targeting export markets. The parameters studied are reflected in Table 3, while Figure 1 shows different research activities during the trial.

**Table 3:** Parameter procedures to be studied in the trial.

S. No.	Parameters	Procedure
1	Milk yield morning	Recorded in kg on the weighing scale at 2 time intervals
2	Milk yield evening	Recorded in kg on the weighing scale at one time
3	Milk fat %	Lactoscan by Milkotronic
4	Milk protein %	
5	Milk lactose %	
6	Milk solids not fats	

### 2.4 Statistical analysis

The data collected were analyzed statistically on SPSS software, the design used was a completely randomized design, one-way ANOVA with repeated measures analysis with independent factors as (feeding, concentrate, and XPM supplementation), and dependent factors as (milk yield and milk composition) was applied. Least significant differences were used as a post-hoc test to compare the differences among the treatment means.

## 3 Results

### 3.1 Milk yield

The data of 12 dairy camels from early to mid-lactation stages (second to fourth parity) based on 04 groups for obtaining the same range of milk production in all groups have been mentioned in Tables 4, 5. Table 4 illustrates milk yield trends for four groups of camels during the 45-day trial period. Group 1 (control) exhibited steady reductions in milk yield, decreasing from 6.70 kg on the first day of the experiment to 4.64 kg on the last day. The average milk of the control group during this period decreased by a drastic 6.16 kg as the animals were shifted from extensive settings to control (semi-intensive settings). Conversely, Groups 2, 3, and 4 demonstrated incremental improvements in production, with Group 4 attaining its peak yield of 8.93 ± 0.74 kg on day 45. The G4 showed a 6.5 kg increase in milk yield on average. The difference in the net change in the milk yield between groups across the trial period was statistically significant from days 16 to 45 (*p*-values < 0.05). However, no significant differences were observed on day 0 (*p* = 0.9968). The findings indicate that feed supplementation and feeding practices in Groups 2, 3, and 4 enhanced milk production relative to the control group. The results particularly highlight that dietary supplements positively influence milk yield in groups receiving additional feed and postbiotics.

**Table 4:** Effects of different diets on camel groups’ milk production at four different times.

Treatments	Day 0	Day 16	Day 30	Day 45
G1 (Control)	6.70 ± 0.32^a^	5.17 ± 0.32^c^	4.84 ± 0.32^b^	4.64 ± 0.32^b^
G2	6.79 ± 0.11^a^	7.11 ± 0.11^b^	7.74 ± 0.11^a^	8.12 ± 0.11^a^
G3	6.72 ± 0.33^a^	7.29 ± 0.33^ab^	7.97 ± 0.33^a^	8.52 ± 0.33^a^
G4	6.77 ± 0.74^a^	7.85 ± 0.74^a^	8.62 ± 0.74^a^	8.93 ± 0.74^a^
P-value	NS	0.0001	0.0002	0.0010

*All pairwise comparisons were performed at a 5% level of significance. Means that having different superscripts are significantly different; NS: non-significant.

**Table 5:** Milk composition (%) of *Barela* camel (*Camelus dromedarius*) with concentrate and postbiotics.

Treatments	Fat		Protein		Lactose		SNF	
	Day 0	Day 45	Day 0	Day 45	Day 0	Day 45	Day 0	Day 45
G1	2.90 ± 0.07	2.82 ± 0.05^b^	3.41 ± 0.04	3.18 ± 0.07	4.79 ± 0.03	4.74 ± 0.04	8.84 ± 0.05	8.61 ± 0.14
G2	3.04 ± 0.07	2.85 ± 0.05^b^	3.46 ± 0.04	3.54 ± 0.07	4.92 ± 0.03	5.12 ± 0.04	8.86 ± 0.05	9.27 ± 0.14
G3	3.09 ± 0.07	3.18 ± 0.05^ab^	3.22 ± 0.04	3.34 ± 0.07	4.89 ± 0.03	4.92 ± 0.04	8.89 ± 0.05	8.97 ± 0.14
G4	2.80 ± 0.07	3.40 ± 0.05^a^	3.48 ± 0.04	3.39 ± 0.07	5.02 ± 0.03	4.83 ± 0.04	9.22 ± 0.05	8.33 ± 0.14
P-value	NS	0.0450	NS	NS	NS	NS	NS	NS

*All pairwise comparisons were performed at a 5% level of significance. Means having different superscripts are significantly different; NS: non-significant.

### 3.2 Milk composition

Table 5 shows the milk composition (fat, protein, lactose, and solids-not-fat) for four feeding treatments (G1, G2, G3, and G4) in camels at days 0 and 45. Non-significant differences were observed between all groups in milk compositions, and they were the same.

Fat percentage showed divergent trends among the groups, with G1 and G2 showing a slight decrease at day 45 (2.90 ± 0.07 to 2.82 ± 0.05 and 3.04 ± 0.07 to 2.85 ± 0.05, respectively), but G3 and G4 with postbiotic supplementation showed an increase, with G4 attaining the highest fat percentage (3.40 ± 0.05) at day 45. Fat increased during the experiment, showing statistically significant differences. The protein content showed consistency across all groups, with slight variations, and the variations across groups were not statistically significant (*p* = 0.3898). G2 exhibited a minor increase (from 3.46 ± 0.04 to 3.54 ± 0.07), but G1 underwent a decrease from 3.41 ± 0.04 to 3.18 ± 0.07. The changes in fat content at day 45 were statistically significant (*p* = 0.045), suggesting an influence from diet or management practices. The amounts of lactose and SNF exhibited fluctuation between days 0 and 45. Group 1 demonstrated slight decreases in lactose (from 4.79 ± 0.03 to 4.74 ± 0.04) and solids-not-fat (SNF) (from 8.84 ± 0.05 to 8.61 ± 0.14), but Group 2 revealed increases in both metrics, with lactose escalating from 4.92 ± 0.03 to 5.12 ± 0.04 and SNF rising from 8.86 ± 0.05 to 9.27 ± 0.14. Notably, G3 and G4 demonstrated steady or marginally variable values for these parameters, with G4 presenting the highest day 0 lactose level (5.02 ± 0.03), which decreased by day 45 (4.83 ± 0.04). The variations in lactose and SNF levels were not statistically significant, since the *p*-values exceeded 0.05. The data indicate changes in milk composition impacted by feeding regimes, with G4 exhibiting greater fat percentage at the trial’s conclusion, while G2 showed an increase in protein and lactose levels. These findings underscore the possibility of targeted dietary measures to improve milk quality components.

## 4 Discussion

In the desert area with less vegetation, camel feeding has greatly shifted toward reliance on supplementary feeding for meeting the animal’s nutritional requirements. While grazing ecosystems are the primary source of sustenance for camels, hence grazing alone is inadequate to fulfill the complete nutrient demands, particularly the mineral needs, of lactating and pregnant camels (30). This highlights the critical role of supplemental feeding in fulfilling nutritional deficiencies, ensuring optimal health, and enhancing milk production in semi-intensive camel management. Semi-intensive systems strike a balance between natural grazing and supplementary feeding, enhancing nutritional balance, productivity, and cost efficiency. All this is dependent on the providence of supplementation to animals and good managerial practices.

In the current scenario of a semi-intensive system, camel feeding becomes progressively dependent on supplements for providing the required nutrients. The wide variation in the constituents of camel milk yield and composition can be attributed to factors such as parity, season, physiological state, geography, and feed (7, 63). However, Abdelgadir et al. (31) showed the non-significant effect of parity on milk constituents. Thus, the inclusion of concentrate and postbiotics likely enhanced the nutritional intake of the camels, leading to improved energy availability for milk production. Camels generally cope with feed scarcity, lactation, and pregnancy by deposition and mobilization as a physiological strategy. Normally, the she-camels utilize their body fats after calving for milk production due to the low feed intake. The camels’ body fat reserves improve in 2–3 months beyond the peak lactation (32).

Regarding seasonal variation, Musaad et al. (67) reported the lowest fat content in July (2.29%), which is lower than our results (2.90%). Similar observations were reported by Igbal (33), which may be due to the dilution effect linked to the production increase (34). Furthermore, the season is a great factor in their variation. While their protein content was lower (2.76%) during October, while our results showed 3.34%. The lactose content in our study was higher (4.90%) in October than in Musaad et al. (67), which was 3.83%. With the advancement of the experiment toward winter, the protein% did not change, but the lactose % gradually decreased.

In our study, the camel parities were second to fourth and were in mid-stage lactation. Normally, the body condition score (BCS) of animals decreases with the parities if the nutritional requirements of the animals are not met properly and as per standards. Hence, it will decrease the camel milk yield and milk composition. Although research has shown that lower BCS of animals at calving did not affect the milk yield, it can lower the milk fat content. Camels that consumed concentrate supplementation may have good body reserves and health status (35). The stage of lactation significantly impacts the protein content in camel milk and is lower at the beginning of lactation (36) but increases significantly during the first 4 months of lactation. The non-significant variation in the milk composition could be due to parity and camels’ body condition scoring. The postbiotics act as antimicrobials and modulate the microbiota and immunity to help the animals toward various metabolic and physiological functions (19). Concentrates and nutrients in XPM provide extra energy to camels by boosting their digestive fermentation, hence producing the building blocks for milk fats in the form of fatty acids. That is why the milk fat content is more flexible and responds quickly to extra energy from supplementation. Along with this, second and third-parity camels usually have a more developed digestive tract for enhanced metabolism, thus can use these supplements more efficiently toward enhancing milk fats. Thus, the provision of concentrate and postbiotics supports higher milk fat without changing the milk protein levels, giving an insight into how camels’ bodies manage milk production in a balanced way. The stability of milk is due to the protein, which supports the animal’s protein needs and strengthens immunity.

Camel milk yield and composition are influenced by a complex interplay of interrelated factors such as parity, management, and lactation stage (37). Our research showed a range of milk yield from 4.84 to 8.93 liters/ day, which is similar to that reported by Faraz et al. (15) that dromedaries can yield approximately 7.38 liters of milk/day under traditional management conditions. Research indicates that adequate nutrition maximizes lactation performance in camels, and supplementing postbiotics may improve the animals’ health, average daily gain, final weight, and milk production (64). Studies have shown that probiotics and their metabolites can positively influence the milk yield by enhancing.

Postbiotics enhance nutrient digestibility and utilization, particularly during periods of high nutritional demand, thereby supporting the critical nutrients needed for production, growth, and absorption. This improved nutrient uptake optimizes feed transformation efficiency in animals (38). Postbiotics also promote gut health and digestive efficiency (39), which further leads to better nutrient utilization. Moreover, postbiotics modulate the intestinal microbiota, fostering the growth of beneficial bacteria such as Lactobacillus and *Bifidobacterium*, while reducing populations of harmful bacteria such as *E. coli* and *Clostridium* spp. (40).

The statistically significant differences in milk yield (*p* < 0.05 from days 16–45) underscore the critical role of strategic dietary supplementation in camel milk production. Our results parallel investigations by Iqbal et al. (65), which demonstrated that precise nutritional management can substantially modify milk production parameters in dromedary camels. Studies have shown that milk yield generally increases from the first to fifth parities, with the highest yields typically observed in the third to fifth parities (41). The remarkable 6.5 liters increase in milk yield in Group 4, with the highest level of XPM supplementation, suggests a potential synergistic effect between concentrate feed and postbiotic interventions. Faraz et al. (16) reported a daily milk yield of 6 liters in first parity camels, increasing to 8.8 liters in the second parity. These results are quite important for sustainable camel farming practices, especially in regions with challenging environmental conditions (68). While Raziq et al. (42) and Ahmad et al. (37) reported the mean daily milk yield in camels to be 8.17 liters in Pakistan. However, as per a study in Sudan, the average daily yield is 4.4 liters/day (43). Comparative analyses of the existing literature reveal the complexity of nutritional interventions in camel milk production. Our study shows clear improvements in milk yield through supplementation. The differential responses observed in Groups 2, 3, and 4 suggest that optimal supplementation strategies may vary depending on the camels’ specific nutritional requirements and environment.

Our studies showed a range of 2.8 to 3.4% fat percentage after supplementing with postbiotics, which are similar to the fat percentage (38.2 ± 10.8 g/L) in whole camel milk; as reported by Konuspayeva et al. (7) and Mati et al. (44). Furthermore, the milk fats remained in both groups at the trial start and day 45. The change in milk fats within all groups remained statistically non-significant. While the milk fat % of 2.5–2.8% has been reported in the Kohi white dromedary camel by Raziq et al. (42), which is lower than that found in the current study. The fat content in camels’ milk can vary between 1.2 and 4.5% (45). However, Park and Haenlein (46) reported that the fat percentage may reach up to 6.4% with the presence of unsaturated and long-chain fatty acids, which helps in lowering the lipid levels in human serum. The same results have been shown by Iqbal et al. (33), with a fat percentage of 3.47–3.68% under Pakistani management conditions. The parity can also affect the fat content of camels. Research showed an increase or no statistically significant differences in fat content with parities, while others showed a decrease in fat content from 5.25% in second parity to 4.69% in third parity in camels (47). Proper management practices during the trial may have reduced stress levels among the camels, contributing to higher productivity. The initial adaptation period allowed the camels to adjust to their feeding regimens, which could explain the significant increases in milk yield observed after this phase.

The results showed a non-significant improvement in milk composition during 45 days’ trial. Results of milk components showed the same range in both groups at days 0 and 45, and could be due to various interrelated factors. One of the main reasons could be that camels were in different parities, and the experimental period is one of the feed scarcities. Another reason could be the non-availability of commercial or balanced rations in remote areas or no fodder or forage availability during winter seasons in desert areas. The feed scarcity due to extreme temperatures can cause animals to be deprived of their basic feed requirements. Hence, the animal’s whole maintenance and productivity are disturbed, and upon supplementation, the animal’s preference is to fulfill their basic maintenance requirements. The supplementation of concentrates provides readily fermentable carbohydrates for enhancing the gut microbial activity and production of volatile fatty acids as acetate and butyrate, the precursors for milk fat synthesis. While the XPM postbiotics improve the gut health and fermentation efficiency, improving the nutrient absorption and energy availability in the camels’ stomach. However, further studies are suggested on a larger number of camels for a longer period of time for validation of these changes.

As per our results, the milk fat as 3.20%, protein 3.43%, lactose 4.93%, and SNF as 8.77%. While Faraz et al. (48) also reported milk fat, protein, lactose, and total solids percentages of 3.88–4.70%, 2.66–4.02%, 3.67–5.04%, and 12.22–14.65% in the Barela camel milk in desert conditions (16). The inclusion of concentrates and postbiotics likely provided essential nutrients that improved milk quality parameters such as fat and protein content (16). This aligns with findings that adequate nutrition is crucial for maximizing lactation performance in camels. The statistically significant changes in fat content (*p* = 0.045) suggest that strategic nutritional interventions can effectively modulate milk biochemical characteristics, potentially offering opportunities for targeted milk production optimization (42). Furthermore, the total solids and SNF may increase with the advancement of lactation, thus fluctuating the milk density throughout the lactation period (47). Additionally, environmental variables, breeds, and analytical procedures contribute to the variability in camel milk constituents as well (49, 50).

As per Park and Haenlein (46) the total protein of camels’ milk varies from 2.15 to 4.90%, while in our research it lies in the same range of 3.18 to 3.54%. The percent of camels’ milk casein to whey protein ratio is relatively lower in camels’ milk than in cows, thus affecting a softer coagulum. Cossins (51) also reported that camels can produce 2.7 times the protein at only 1.3 times the dry matter as required by cattle. The presence of beneficial compounds from postbiotics can influence metabolic pathways associated with milk synthesis. The adaptation phase allowed camels to adjust to new feeding regimens, potentially enhancing their ability to utilize nutrients effectively for milk production (33). Research have shown that protein content increases with parity (47). A study in Sudan showed a reduction in protein content in camel milk from traditional pasture (43). Another study showed a lower protein percentage in second parity camels (3.60%) than in third parity (3.46%) (43).

The lactose percentage in our study was observed to be in the range of 4.74–5.12%, which is similar to the values reported by 5.15% (37). As per Devendra et al. (45), lactose content is stable and ranges from 3.5 to 4.5%. Mustafa et al. (47) reported that lactose varies with season and decreases with increasing parity. Lactose is the main carbohydrate promoting the formation of *a Bifidobacterium* environment for the development of the nervous system (46). Postbiotics may enhance gut health and nutrient absorption, leading to improved milk composition (52). Variations in environmental conditions can also affect milk composition; however, controlled management practices during the trial helped mitigate these effects.

This probiotic approach demonstrates potential for improving the nutrient digestibility of crude protein and total digestible nutrients, promoting gut health, and boosting the resistance to infections in camels (53). This enhancement resulted in elevated dry matter intake and voluntary water consumption, facilitating improved nutrition absorption and hydration, particularly during extended working circumstances (52). Postbiotics are characterized as non-viable microbial metabolites or metabolic byproducts that provide health advantages to the host. They are essential for boosting gut health and optimizing metabolic activities, hence significantly affecting growth performance through improved nutrient absorption and metabolic health indices (69). This study emphasizes the potential of utilizing lactic acid bacteria from camels as probiotics, which may enhance health by supporting the camels’ microbiome (54).

The incorporation of postbiotics into the dietary regimen of dromedary camels confers numerous health advantages. Increased gut microbial diversity correlates with enhanced digestion and nutrient absorption, essential for sustaining good health in semi-intensive feeding regimes. Postbiotic supplementation may also influence immunological responses, potentially decreasing the prevalence of illnesses typically seen in intensively managed herds (55). Moreover, research has demonstrated that postbiotics can mitigate oxidative stress and enhance metabolic profiles in animals subjected to intense feeding protocols (66). The composition of camel milk, specifically its protein, fat, and total solid content, seems to be influenced by the use of postbiotics and rigorous feeding methods. However, more studies are needed for confirmation of changes in milk composition and yield. Research indicates that augmenting camels’ diets with supplementary protein can result in elevated amounts of both milk fat and protein. This may enhance the nutritional quality of camel milk for human consumption, which is advantageous for places that depend significantly on it (56).

The comparative analyses with existing literature underscore the complexity of camel milk composition modifications and variations depending on production systems, locations (60), availability of water and differences in feed types. While our study showed relatively stable protein content across all treatments, and relatively improved with parities. Mati et al. (44) reported that protein can vary more under different nutritional interventions. The fat percentage in camels’ milk normally increases beyond peak lactation by 2–3 months, and by supplementation. The observed increases in lactose and solids-not-fat (SNF) in Group G2, coupled with the marginal changes in other groups, indicate that specific nutritional combinations can differentially impact milk composition. These findings contribute to the growing body of research demonstrating the potential for precision nutrition in camel dairy systems, offering insights that could be valuable for producers targeting specialized markets, particularly in regions such as the Gulf and China (57, 58). As this is a short study, more treatment groups, an increased number of animals, a longer experimental period, and various seasons can confirm these preliminary results and also reveal more interesting outputs.

This is a pilot study, and before testing something new on many animals it is important to do a pilot. Hence, the study was limited by the small number of animals, in different parities, and the fact that the animals were grazing in a desert area. Notwithstanding the limitations, it contributes valuable knowledge for enhancing sustainable camel farming practices while also addressing the growing global demand for camel milk. Thus, further studies are needed as there are some limitations as a low number of animals available for study, confinement to a particular pasture because of the difficulty of performing a field study in camels, the study’s duration, and to check different weather effects. Notwithstanding these limitations, this pilot study has increased the knowledge of enhancing camel milk production and welfare. Future research should focus on comprehensive economic assessments to clarify the financial implications and investigate the long-term effects of these nutritional strategies on camel productivity and welfare perspective that is very important. Expanding research to include various breeds, larger sample sizes, and diverse environmental conditions could provide deeper insights into improvement in camel milk yield and quality.

## 5 Conclusion

This pilot study tested feed integration of grazing systems in dairy camels and revealed that dietary supplementation, particularly with higher levels of XPM, led to a significant increase in milk yield, with one group improving milk production up to a 6.5-liter increase. While milk fat content ranged from 2.8 to 3.4% after supplementation and was improved significantly by postbiotics and supplementation, milk protein remained the same. However, lactose and solids-not-fat contents did show increases in specific groups. These improvements are attributed to postbiotics enhancing gut health, nutrient absorption, and metabolic activities in camels. Our findings suggest that supplementation with postbiotics can improve milk production and quality, promoting the animal’s wellbeing. Further studies are needed to confirm our preliminary results.

## Data Availability

The original contributions presented in the study are included in the article/supplementary material, further inquiries can be directed to the corresponding authors.
